# (Un)Forbidden Words: Diversity, Equity & Inclusion in Gerontology & Geriatrics Education

**DOI:** 10.1093/geroni/igaf122.687

**Published:** 2025-12-31

**Authors:** Rona Karasik, Mark Brennan Ing

## Abstract

Gerontology and geriatrics have long advocated for the consideration and inclusion of older adults in community, policy, practice and research. Systemic ageism, however, cannot be separated from the context and power dynamics inherent in structural racism, sexism, heterosexism, ableism, xenophobia and the like. Understanding aging requires consideration of the entire life course, including the social determinants that impact the lives and experiences of all older adults. So how do gerontological educators proceed as higher education grapples with directives determined to dismantle scientific inquiry in general, and exploration of diversity, equity and inclusion in particular? This symposium, sponsored by Gerontology & Geriatrics Education, reflects on current challenges and considers pathways forward as we continue to advocate for diversity, equity and inclusion in gerontological education. First, as an introduction, Karasik & Kishimoto discuss the concepts of diversity, equity and inclusion as they relate to gerontology and geriatrics education. Next, Kishimoto and Karasik consider the role of antiracist pedagogy in gerontological education and how it can be applied to teaching about health disparities throughout the life course and across generations. Third, Montepare and colleagues address the concept of age inclusivity as perceived by students/faculty/staff and its implications for educational efforts. Fourth, Flatt highlights current obstacles and ways for education, training, and activism to support sexual gender and minority aging research to move forward. Finally, discussant Brennan-Ing will engage participants in a structured discussion of next steps for defending diversity, equity, and inclusion as the essence of gerontological inquiry and education.

